# Factorization of joint metacommunity diversity into its marginal components: an alternative to the partitioning of trait diversity

**DOI:** 10.1007/s12064-020-00316-4

**Published:** 2020-06-01

**Authors:** Hans-Rolf Gregorius

**Affiliations:** 1 Abteilung Forstgenetik und Forstpflanzenzüchtung, Universität Göttingen, Büsgenweg 2, 37077 Göttingen, Germany; 2 Institut für Populations- und ökologische Genetik, Am Pfingstanger 58, 37075 Göttingen, Germany

**Keywords:** Factorization, Joint diversity, Marginal diversity, Effective numbers, Partitioning of diversity, Differentiation, Polymorphism

## Abstract

Diversity in metacommunities is traditionally viewed to consist of the diversity within communities ($\alpha$) that is complemented by the differences between communities ($\beta$) so as to result in the total diversity ($\gamma$) of the metacommunity. This perception of the partitioning of diversity, where $\beta$ is a function of $\gamma$ and $\alpha$ (usually $\beta =\gamma /\alpha$ with all components specified as effective numbers), has several drawbacks, among which are (1) $\alpha$ is an average that can be taken over communities in many ways, (2) complete differentiation among communities cannot always be uniquely inferred from $\alpha$ and $\gamma$, (3) different interpretations of $\beta$ as effective number of communities (e.g., distinct or monomorphic) are possible, depending on the choice of ideal situations to which the respective effective numbers refer, and (4) associations between types (species, genotypes, etc.) and community affiliations of individuals are not explicitly covered by $\alpha$ and $\gamma$. Item (4) deserves special regard when quantifying metacommunity diversity. It is argued that this requires consideration of the joint distribution of type-community combinations together with its diversity (joint diversity) and its constituent components: type and community affiliation. The quantification of both components can be affected by their association as realized in the joint distribution. It is shown that under this perception, the joint diversity can be factorized into a leading and an associated component, where the first characterizes the minimum number of communities required to obtain the observed joint diversity given the observed type distribution, and the second specifies the effective number of types represented in the minimally required number of communities. Multiplication of the two yields the joint diversity. Interchanging the roles of community and type, one arrives at the dual factorization with leading minimum number of types and associated effective number of communities. The two dual factorizations are unambiguously defined for all measures of diversity and can be used, for example, to indicate structural characteristics of metacommunities, such as type differentiation among communities and associated type polymorphism. The information gain of the factorization approach is pointed out in comparison with the classical and more recent modified approaches to partitioning total type diversity into diversity within and between communities. The use of factorization in analyses of latent community subdivision is indicated.

## Introduction

Diversity in metacommunities is traditionally viewed to consist of the diversity within communities that is complemented by the differences between communities so as to result in the total diversity of the metacommunity (among the first is Whittaker 1960). In established terminology, these three levels of variation are denoted as $\alpha$- (within communities), $\beta$- (between communities) and $\gamma$-diversity (total metacommunity). This view concentrates on trait (mostly genetic or species) diversity and the way it is distributed in a metacommunity with the focus on possibilities of partitioning the total trait diversity into a component covering the diversity within communities and a component reflecting the differences between communities.

As was pointed out by a number of authors, the crucial component is $\alpha$-diversity (for an overview, see, e.g., Chiu et al. 2014). Besides uniqueness problems (a virtually indefinite number of “averages” of diversities within communities exist that fulfill the partitioning criterion; Gregorius 2014), it is known for its failure to account for aspects of differentiation among communities in combination with $\gamma$-diversity (see, e.g., Jost et al. 2010). The latter in turn implies ambiguity in interpretation of $\beta$-diversity to reflect differences between communities when considered as a function of $\alpha$ and $\gamma$-diversity only. It was shown by Gregorius (2010) that the reason lies primarily in the neglect of effects on diversity that are explicitly due to community affiliation and its association with the trait. This phenomenon was indicated in earlier work, e.g., by Routledge (1979), Jost (2008), or Tuomisto (2010).

### The metacommunity context

Indeed, the characterization of metacommunity members by their community affiliation is rarely an explicit object of studies of variation or diversity. Numbers and sizes of communities, for example, are not expressly considered as diversity generating factors. Yet, communities may be characterized in different ways, many of which refer to functional characteristics, environmental conditions, geographic region and the like, all of which apparently specify other properties for which individuals can be distinguished and display diversity. Hence, metacommunity diversity is intrinsically a joint kind of diversity that encompasses both a distinguished trait and community affiliation as flanking sources of diversity. When problems of ecological stability are studied with respect to ecological diversity, the obvious main subject of analysis is neither species (or genetic) nor environmental diversity alone but rather the combination of both.

The effect of this combination on (joint) diversity becomes apparent through the associations between the two components “trait” (genetic, species, etc.) and “community affiliation” (environment, social rank, etc.). Associations rank between complete association (where, e.g., communities are completely differentiated for their trait states or types, for short) and the absence of association (where, e.g., types show equal distribution within all communities). Particularly complete association underlines the role played by the “flanking” or “marginal” distributions, since in this case, the joint diversity equals the overall (marginal) trait diversity (when differentiation of communities for types is complete) or the diversity of communities (when differentiation of types for community affiliation is complete, see Gregorius 2010). The marginal distributions therefore set bounds that are essential for the assessment of joint diversities.

Consequently, in the present paper, attempts to decompose, or factor, diversity into components will be targeted toward the joint diversity, where the marginal distributions are considered as interacting components. This contrasts with the classical forms of partitioning diversity which focus on the type diversity in the total metacommunity as compared with the type diversity within the constituent communities. To relate to common approaches as summarized, e.g., in the above-cited work of Jost (2008) and Gregorius (2016), appropriate effective numbers for the components of diversity and their interaction in producing the target diversity will be derived. Hereby, ambiguity inherent in the conception of effective numbers will be taken special care of and an unambiguous alternative will be presented.

### The differentiation problem

Such ambiguity includes reference to effective numbers of communities in terms of their “distinctness” and thus differentiation especially in defining $\beta$-diversity (see, e.g., Jost 2007). Intrinsically, community differentiation addresses differences in type distribution between communities without implying any statements about the diversities realized in the communities (Gregorius 2016). To avoid misconception, a distinction is made by occasionally adding the adjective “compositional” to differentiation, i.e., compositional differentiation. Albeit, the compositional extremes of complete absence and complete presence of differences in type distribution between communities affect relations among components of diversity as well, however, without determining the sizes of the components.

When treating effective numbers of “distinct” communities in diversity analyses, it is therefore crucial to make clear that these numbers do not specify a number of completely differentiated communities realized in a metacommunity (compare the example of Table 2, where communities are clearly differentiated among each other for their types, but none of the communities is completely distinct). They rather make a statement about how many communities an assemblage of completely differentiated communities (the ideal situation) it would require to generate the observed diversity. On the other hand, ideal situations can be chosen in different ways, and this may lead to different interpretations of the same formal definition of $\beta$-diversity. An example is provided in the paper of Gregorius (2016), where it is argued that the perception of $\beta$ as an effective number of distinct communities can be modified into a “number of effectively monomorphic communities.”

### Measures of diversity

More fundamentally, it might be worth emphasizing that most measures of diversity have a biological interpretation that is not explicitly specified in terms of the number of types involved in the biological process. Simpson’s diversity in its version as the probability of sampling without replacement two individuals of different type, for example, finds a biological interpretation in terms of species interactions depending on the frequency of interspecific encounters. The number of species participating in the encounters is not of primary interest here. Yet, the measure allows for determination of an effective number by equating the observed diversity to the diversity resulting for a number of equally frequent types and solving for this number. This relates to a more elementary level of interpreting effective numbers in that it touches on the concept of diversity.

It applies to all admissible diversity measures and it informs about the number of types “effectively” participating in the biological process. Without explicit reference to the underlying, biologically motivated diversity measure, such effective numbers make only abstract numerical and scale-free statements that provide limited information on biologically relevant characteristics. Rényi-diversities (see bottom of Table 1), which are admissible diversity measures that equal their effective numbers, are an example. They may be obtained as an effective number from quite a variety of primary diversity measures (Jost 2007; Gregorius 2009, 2014)^1^. Diversity effective numbers simply transform the interpretable diversity measure into the number of types effectively determining the original diversity measure.

**Table 1:** Notations

$q_{i,j} :=$ joint relative frequency of the *i*th type with *j*th community affiliation ($i=1,\ldots ,N_T$, $j=1,\ldots ,N_C$, $\sum _{i,j}q_{i,j}=1$). The array of $q_{i,j}$s is denoted by ${{\varvec{\mathsf{{ q}}}}}$	
$p_i:= \sum _jq_{i,j}$ or the relative frequency of the *i*th type in the total metacommunity (marginal type frequencies, array—or vector—denoted by ${{{\varvec{\mathsf{{ q}}}}}}_T$)	
$c_j:=\sum _iq_{i,j}$ or proportion of individuals belonging to the *j*th community (marginal community frequencies or sizes, array—or vector—denoted by ${{{\varvec{\mathsf{{ q}}}}}}_C$)	
${{{\varvec{\mathsf{{ q}}}}}}^N_C:=$ joint frequency array ${{{\varvec{\mathsf{{ q}}}}}}'$ derived from array ${{\varvec{\mathsf{{ q}}}}}$ by setting $q_{i,j}'=p_i /N$, where the $p_i$s are the marginal type frequencies in ${{\varvec{\mathsf{{ q}}}}}$ and $j=1,\ldots ,N$	
${{{\varvec{\mathsf{{ q}}}}}}^N_T:=$ joint frequency array ${{{\varvec{\mathsf{{ q}}}}}}'$ derived from array ${{\varvec{\mathsf{{ q}}}}}$ by setting $q_{i,j}'=c_j /N$, where the $c_j$s are the marginal community frequencies in ${{\varvec{\mathsf{{ q}}}}}$ and $i=1,\ldots ,N$	
$v({{{\varvec{\mathsf{{ u}}}}}}):=$ diversity measure *v* applied to the frequency array or vector ${{\varvec{\mathsf{{ u}}}}}$	
$v_{TC}:= v({{{\varvec{\mathsf{{ q}}}}}})$ or joint diversity of trait *T* and community *C* for joint frequency array ${{\varvec{\mathsf{{ q}}}}}$	
$v_T:=v({{{\varvec{\mathsf{{ q}}}}}}_T)$, or diversity of the marginal type frequencies	
$v_C:=v({{{\varvec{\mathsf{{ q}}}}}}_C)$, or diversity of the marginal community frequencies	
$v^e:=$ the diversity effective number of the diversity measure *v* (results as the number of entities of equal frequency for which the diversity measure *v* equals the observed diversity; for the extension to real numbers of entities, see Appendix II in Gregorius 2014)	
*Rényi-diversities*, also called *Hill numbers*, are diversity measures of the form $\big (\sum _ku_k^a\big )^{1\over 1-a}$, $u_k>0$, $\sum _ku_k=1$, $a\ne 1$. The limit $a\rightarrow 1$ exists as $\exp (\sum _ku_k\cdot \ln u_k)$	

The wide range of diversity measures with substantial biological interpretation also justifies to not insist on specific properties frequently stipulated for diversity measures and particularly for their effective numbers (such as the replication principle, see, e.g., Ricotta and Szeidl 2009). A typical category of diversity measures whose effective numbers do not comply with the replication principle is characterized by the above-mentioned probability models of non-random encountering (see, e.g., Patil and Taillie 1982, or Gregorius 2009). Another category that has wide application in physics and economy relates to non-independent information content.

To allow for this range, diversity measures are considered admissible if they fulfill not more than the basic evenness criterion (diversity increases as the difference in frequency between two types decreases while the sum of their frequencies remains the same). The criterion is also known as the principle of transfers (see, e.g., Patil and Taillie 1982). The major results obtained in the present paper do not require more than the evenness criterion and are therefore not restricted to special types or categories of diversity measures (even though translation into Rényi-diversities will be provided in each case for habitual reasons).

### Factorization of joint diversity

In the following, it will be shown that within this wide scope of measuring diversity, joint diversities allow for multiplicative decomposition (factorization) of their effective numbers into components relating to the marginal variables “trait” and “community affiliation.” Herewith, one component (the leading component) is distinguished by realizing a minimization criterion. As applied to community affiliation as leading component, for example, the criterion determines the minimum number of communities required to realize the observed joint diversity given the observed trait distribution. The criterion does not rely on the concept of effective numbers at the outset, but it is amenable to an interpretation in terms of such numbers. The minimally required number lays the basis for a unique factorization of the joint diversity into the leading (e.g., community affiliation) and the associated marginal component (trait). The relation of the factorization to a more recently proposed method of partitioning diversity is demonstrated that avoids the arbitrariness inherent in averaging diversities within communities (Chiu et al. 2014).

## Number of communities for given marginal type frequencies

Particularly for large numbers of individuals, their types can be distributed over several numbers of communities without affecting the overall type frequencies. As a result, the joint diversity should increase roughly with the number of communities which the individuals are assigned to. However, if the assignment is such that individuals of the same type never appear in different communities (complete community differentiation), the joint diversity equals the overall (marginal) type diversity ($v_{TC}=v_T$), since the number of different type-community combinations equals the number of types. Thus, each community is represented in only one type, and the joint diversity is not affected by such assignments to communities.

Apparently, this changes only after individuals of the same type are assigned to different communities, so that the number of different type-community combinations increases with the number of communities that are represented in each type. Moreover, the more even a type is distributed over communities the larger the joint diversity by the evenness criterion. For a given number of communities, the maximum diversity is then reached if in each type all communities are represented and are so at equal proportions. At this end, types are not differentiated for their community affiliations, and in consequence communities are not differentiated for their types. While during this equalization process the type frequencies remain the same, an initial community differentiation is gradually lost.

In this context, the diversity of the observed joint frequency array ${{{\varvec{\mathsf{{ q}}}}}}=\{q_{i,j }\}$ is to be related to the diversity of an array ${{{\varvec{\mathsf{{ q}}}}}}'$ of the form ${{{\varvec{\mathsf{{ q}}}}}}'={{{\varvec{\mathsf{{ q}}}}}}^N_C$ with $q'_{i,j}=p_i/N$ (see Table 1). As indicated above, the $q'_{i,j}$s reflect the situation where there is no differentiation for types among communities, and communities have equal shares within each type and consequently in the total metacommunity. The (joint) diversity of this modified array, i.e., $v({{{\varvec{\mathsf{{ q}}}}}}^N_C)$, will be denoted by $v_{TC}'$ for the moment. For $N=N_C$, it then follows from the evenness criterion that $v_{TC}\le v_{TC}'$.

$v_{TC}'$ is a strictly increasing function of *N* for given marginal type frequencies, and it starts with $v_T$ for $N=1$ and has a unique solution $N=N_C^{\circ }$ for which $v_{TC}'=v_{TC}$. Since $v({{{\varvec{\mathsf{{ q}}}}}}^{N_C}_C )\ge v_{TC}$, it always holds that $N_C^{\circ }\le N_C$. Note that for equally sized communities, $N_C^{\circ }=N_C$ does not hold when communities differ for their type frequencies.

The most characteristic property of $N_C^{\circ }$ becomes apparent when considering joint distributions which share their marginal type frequencies and have equal joint diversities. All of these distributions have the same $N_C^{\circ }$, but they may differ in their $N_C$ values. By the above result, all of these $N_C$ values are equal to or greater than $N_C^{\circ }$. Hence, the conclusion that for the observed marginal type frequencies, $N_C^{\circ }$ is the *minimum number of communities required to obtain the observed joint diversity*. In an earlier attempt of finding effective numbers of communities based on joint diversities (Gregorius 2010, section 10), this minimization characteristic escaped notice.

In a strict sense, however, $N_C^{\circ }$ should be a number of communities and therefore should be a natural number that makes ${{{\varvec{\mathsf{{ q}}}}}}_C^{N_C^{\circ }}$ a proper joint frequency array to which the diversity measure *v* can be applied. As was shown by Gregorius (2014, Appendix II) the diversity of uniform frequency arrays can always be monotonically extended to real numbers. To maintain the relationship to natural numbers, it is, however, essential to consider the largest natural number *N* for which $v({{{\varvec{\mathsf{{ q}}}}}}^{N}_C)\le v_{TC}$ and to realize that necessarily $N\le N_C^{\circ }<N+1$ for this number *N*.

For Rényi-diversities (where $v=v^e$), $v({{{\varvec{\mathsf{{ q}}}}}}^N_C)=N\cdot v({{{\varvec{\mathsf{{ q}}}}}}_T)=N\cdot v_T$, which, by setting $N\cdot v_T=v_{TC}$, yields $N_C^{\circ }=v_{TC}/v_T$. This family of diversity measures now allows $v_{TC}/v_T$ to be interpreted as specifying the minimum number of communities required to realize the observed joint diversity when retaining the observed marginal type frequencies.

Since both, $N_C^{\circ }$ and the marginal effective number $v_C^e$ of communities refer to numbers of communities, comparison between the two quantities is in demand. At least in the example provided for Rényi-diversities in Table 2, it becomes apparent that, in accordance with the minimum characteristic of $N_C^{\circ }$, distinctly fewer communities are required to obtain the observed joint diversity that are effectively represented in the marginal community composition ($N_C^{\circ }\ll v_C^e$). However, more analysis might be desirable to assess the share in this reduction that is due to the comparatively high proportion of type-community combinations (5/12) not represented in the example.

**Table 2:** A fictitious numerical example demonstrating for several orders *a* of Rényi-diversity the diversity components $v_T$, $v_C$, $v_{TC}$, the minimally required numbers of communities ($N_C^{\circ }$) and types ($N_T^{\circ }$) as well as their associated effective numbers of types ($N_{T<C}^{e}$) and communities ($N_{C<T}^{e}$), respectively, and the dual $\beta$-diversities $N_C/N_C^{\circ }$ and $N_T/N_T^{\circ }$

Joint and marginal frequencies									
	$C_1$	$C_2$	$C_3$	$\sum _T$					
$T_1$	0.18	0.00	0.28	0.46					
$T_2$	0.00	0.08	0.09	0.17					
$T_3$	0.00	0.00	0.20	0.20					
$T_4$	0.09	0.08	0.00	0.17					
$\sum _C$	0.27	0.16	0.57	1.00					

Diversity components, minimum numbers, effective numbers and $\beta$*-diversities*									
Order	$v_T^e$	$v_C^e$	$v_{TC}^e$	$N_C^{\circ }$	$N_{T<C}^{e}$	$N_T^{\circ }$	$N_{C<T}^{e}$	${N_C\over N_C^{\circ }}$	${N_T\over N_T^{\circ }}$
$a=0$	4.000	3.000	7.000	1.750	4.000	2.333	3.000	1.714	1.714
$a=1$	3.602	2.630	6.200	1.721	3.602	2.357	2.630	1.743	1.697
$a=2$	3.232	2.362	5.562	1.721	3.232	2.355	2.362	1.743	1.699
$a=\infty$	2.174	1.754	3.571	1.643	2.174	2.036	1.754	1.826	1.965

### Interpretation of $N_C^{\circ }$ as an effective number

Despite its definition as minimally required number of communities, $N_C^{\circ }$ can also be conceived of as an effective number on the basis of the above demonstrations. According to its general concept (see, e.g., Gregorius 2016, Appendix A), determination of an effective number requires specification of an ideal situation and a characteristic variable associable with a target variable. In the present case, the target variable is the number of communities, the ideal situation is specified by the joint frequency arrays ${{{\varvec{\mathsf{{ q}}}}}}^N_C$ with natural *N*, and the characteristic variable is made up of two components, with the primary component given by the joint diversity and the secondary component given by the type frequencies. From the above demonstrations, it follows that for each observed joint frequency array ${{\varvec{\mathsf{{ q}}}}}$, there exists an ideal array ${{{\varvec{\mathsf{{ q}}}}}}^N_C$, with *N* extendable to a real value that implies equality between the observed and the ideal array for each of the two characteristic variables. Moreover, any two ideal distributions with equal characteristic variable are identical for their target variable $N_C$. Hence, the number $N_C^{\circ }$ can be conceived of as both the result of a minimization process and an effective number.

To emphasize the difference from other effective numbers of communities like $\beta$-diversity, it seems appropriate at this place to recall that they do not result from an optimization process. Instead, $\beta$-diversity is claimed to make structural statements concerning the (effective) number of “distinct” communities (which altogether is critical as mentioned above). In contrast, $N_C^{\circ }$ cannot be used to directly quantify structural aspects such as distinctness or its opposite. In particular, $N_C^{\circ }$ is not aimed at providing an effective number of “undifferentiated” communities as one might be tempted to conclude from its derivation. The fact that $N_C^{\circ }=1$ only for $v_{TC}=v_T$, which holds only for completely differentiated communities and irrespective of the type diversity within the communities, makes this evident. At the same time, however, this fact indicates possible relations of $N_C^{\circ }$ to structural aspects as will be elaborated in the fifth section.

A feature of $N_C^{\circ }$ that distinguishes it from most common approaches to effective numbers is to be seen in its ideal situation and characteristic variable, which explicitly include information on the observed data in terms of the marginal type frequencies. In common approaches, the ideal reference is usually almost completely abstracted from the observation by assumptions like complete community differentiation, equal diversities for all communities including uniform type distributions, and equal community sizes. Obviously, the higher the degree of such abstraction, the more problems arise in relating the interpretation of the resulting effective numbers to observed properties. Effective numbers of “distinct” communities are an example, as is mentioned above. Since $N_C^{\circ }$ directly quantifies a property of the observed frequency array without drawing on an idealized array, its perception as minimally required number should be given preference over its interpretation as an effective number of communities.

## Factorization of joint diversity into its type and community component

Diversity measures do not distinguish between the quality of the entities for which they are obtained. Thus, for a specified diversity measure, its values may cover the same range when applied to the distribution of a single qualitative variable or to multiple variables. For multiple variables, the entities are just combinations of states of the involved variables. The same applies to the corresponding diversity effective numbers of entities. For the present case of the two variables “type” and “community affiliation,” there may exist type-community combinations with zero frequency in the joint distribution, even though all marginal type and community frequencies are positive (for a fictitious example see Table 2). By analogy with the diversity effective number of a single variable, for determination of the joint diversity effective number $v_{TC}^e$, only numbers of equally frequent combinations matter that do not exceed the number of combinations with positive frequency. The involved marginal numbers of types and communities do not explicitly enter the calculations.

Hence, joint diversities $v_{TC}$ and their effective numbers $v_{TC}^e$ do not depend on the numbers of states represented in the two marginal variables (type and community). On the other hand, the marginal variables set the upper limit to the joint diversity in that $v_{TC}^e\le N_T\cdot N_C$ with equality only if all of the $N_T\cdot N_C$ potential(!) combinations appear at equal (positive) frequencies. Therefore, particularly if several potential combinations have zero frequency, the question arises as to the number of types $N_T'$, say, and the number of communities $N_C'$, say, which actually or effectively participate in creating the observed joint diversity. Clearly, $N_T'\le N_T$ and $N_C'\le N_C$.

Participation in turn requires specification of the mode according to which the numbers contribute to the joint distribution. As indicated above, the full potential of contributions is always realized under independent and equal participation of the marginal variables in the joint frequency array. Setting this as the ideal situation on which effective numbers are based, one arrives at a joint diversity the effective number of which (ideally) equals the product $N_T'\cdot N_C'$. The components of the product are the natural candidates for any attempt of factorizing the joint diversity. Factorization must therefore be performed for the effective number of the joint diversity under consideration

Consequently, equating the product with the observed joint diversity effective number $v_{TC}^e$, i.e., $v_{TC}^e=N_C'\cdot N_T'$, would set the range within which the numbers $N_C'$ and $N_T'$ were allowed to vary. While the solution of this equality always exists and is unique with respect to the product $N_C'\cdot N_T'$ of real numbers, uniqueness of the two components is not achieved until one of them is determined via desirable properties. Determination of any of the two factors then settles the other, and both must unambiguously refer to the marginal variables.

The latter fact asks for distinction between a leading and an associated component of the factorization, where the leading component reflects the desirable properties. The above demonstrations suggest *C* as a leading component, for which the number $N_C^{\circ }$ of communities reflects a desirable property via its minimization characteristic. This justifies the choice of $N_C'=N_C^{\circ }$, which in turn determines the “associated effective number” $N_T'$ of types as $v_{TC}^e/N_C^{\circ }$. The notation $N_{T<C}^{e}:=v_{TC}^e/N_C^{\circ }$ will be used to emphasize the associative character of this effective number as well as the *community-centered perspective* of the factorization. One then arrives at the multiplicative decomposition (or factorization) of joint diversity in the form

$$\begin{aligned} v_{TC}^e=N_C^{\circ }\cdot N_{T<C}^{e}\end{aligned}$$

Indeed, $N_{T<C}^{e}$ is to be addressed as an effective number, since its specification (as opposed to $N_C^{\circ }$) depends on idealizing assumptions on joint distributions.

For Rényi-diversities (see bottom of Table 1), where $N_C^{\circ }=v_{TC}^e/v_T^e$, one obtains $N_{T<C}^{e}=v_{TC}^e/N_C^{\circ }=v_T^e$. Here, the associated effective number of types equals the marginal type diversity and is therefore independent of the (effective) number of communities as well as their associations with the types. Effects of associations are therefore completely covered by $N_C^{\circ }$.

This property of Rényi-diversities need not apply to other measures of diversity such as given in Introduction. To determine the limit for the associated component $N_{T<C}^{e}$, let *N* again be the largest natural number with $v({{{\varvec{\mathsf{{ q}}}}}}^N_C)\le v_{TC}$. Then, $N\le N_C^{\circ }<N+1$ and $v({{{\varvec{\mathsf{{ q}}}}}}^N_C)\le v_{TC}<v({{{\varvec{\mathsf{{ q}}}}}}^{N+1}_C)$. The smallest possible value for $N_C^{\circ }$ given $v_{TC}$ would be $N_C^{\circ }=N$, for which one obtains $N_{T<C}^{e}\cdot N_C^{\circ }=v_{TC}^e=v^e({{{\varvec{\mathsf{{ q}}}}}}^N_C)\le N\cdot N_T\le N_C^{\circ }\cdot N_T$, from which $N_{T<C}^{e}\le N_T$ follows. Since $v_{TC}^e=N_{T<C}^{e}\cdot N_C^{\circ }$, the smallest possible value for $N_C^{\circ }$ specifies the largest possible value for $N_{T<C}^{e}$ for given $v_{TC}^e$, and this implies that always $N_{T<C}^{e}\le N_T$. In summary

$$\begin{aligned} N_C^{\circ }\le N_C \hbox { and } N_{T<C}^{e}\le N_T \end{aligned}$$

which concurs with intuitive expectations and conceptual reasoning.

To appreciate the information provided by the associated effective number of types it is helpful to recall that joint frequency arrays for which $N_C^{\circ }$ is realized are characterized by communities of equal size and type distributions that are the same within each community and therefore equal the marginal type distribution. One thus expects $N_{T<C}^{e}$ to be given by $v_T^e$ as is true for Rényi-diversities. Yet, for other categories of diversity measures such as addressed in Introduction, this does not apply (checked numerically by the author for the array in Table 2). An explanation is suggested by the difference in the biological reasoning of the categories referring to random and non-random encounters of community members. Besides relating to marginal type diversities, the associated effective number of types is apparently sensitive to constructional differences in categories of diversity measures.

More practically relevant properties will be treated later on with reference to structural characteristics of joint distributions. For the time being, it may suffice to recall that $N_C^{\circ }$ refers to a hypothetical array of joint frequencies that shares its marginal type distribution and diversity with the observed joint distribution. Therefore, $N_{T<C}^{e}$*can be understood to quantify the effective number of types represented in the minimally required number of communities*.

## The dual analogue: the type-centered perspective

The apparent asymmetry in the above treatment of the community and type component *per se* asks for consideration of its reversal. In other words, a transition from the community-centered to a type- or trait-centered perspective is suggested. Given the marginal distribution of community affiliations (community sizes), one would then aim at finding the minimum number of types required to obtain the observed joint diversity in the first place. In this way, the contribution of types to the ecological diversity of a metacommunity is assessed on the basis of the observed partition of the metacommunity into its constituent communities. This done, the associated effective number of communities is to be specified. Since community determinants are frequently considered to be less dynamic than the traits of the community members existing on or adapting to these determinants, this may be the more relevant perspective to be taken in many ecological studies.

In essence, this perspective simply marks the dual analogue of the previous community-centered approach to the decomposition of joint diversity into community and type components. It therefore results from just reverting the roles of *T* and *C*. All of the conclusions obtained so far apply analogously. Thus, one first determines the minimum number $N_T^{\circ }$ of types required to obtain the observed joint diversity as the solution $N=N_T^{\circ }$ for which $v({{{\varvec{\mathsf{{ q}}}}}}_T^N)=v_{TC}$. This is followed by specifying the associated effective number $N_{C<T}^{e}$ of communities from

$$\begin{aligned} v_{TC}^e=N_{C<T}^{e}\cdot N_T^{\circ }\end{aligned},$$

i.e., $$N_{C<T}^{e}=v_{TC}^e/N_T^{\circ }$$. Now, $$N_T^{\circ }\le N_T$$ and $$N_{C<T}^{e}\le N_C$$.

For Rényi-diversity, $N_T^{\circ }=v_{TC}^e/v_C^e$, and thus, $N_{C<T}^{e}=v_C^e$. It is worth noting that none of the two components resembles any of the effective numbers involved in the classical partitioning of $\gamma$- into $\alpha$- and $\beta$-diversity.

## Concluding remarks

The minimum number $N_C^{\circ }$ of communities required to obtain the observed joint diversity is a parameter that provides no direct information on structural characteristics relating, for example, to the distribution of diversity over communities. Yet, $N_C^{\circ }$ can be transformed into an *indicator of structural characteristics* under the community-centered perspective when considering the deviation of $N_C^{\circ }$ from $N_C$. To see this, consider that $N_C^{\circ }=N_C$ is equivalent to $v({{{\varvec{\mathsf{{ q}}}}}}_C^{N_C})=v_{TC}$, which in turn is equivalent to ${{{\varvec{\mathsf{{ q}}}}}}_C^{N_C}={{{\varvec{\mathsf{{ q}}}}}}$. The latter follows directly from the specification of $N_C^{\circ }$ via equalization of the community representations within the types (see also third paragraph in the second section). Therefore, $N_C^{\circ }=N_C$ only if the same communities are represented at equal frequencies within each type. From a more familiar perspective, this is equivalent to “$N_C^{\circ }=N_C$ only if communities are not differentiated for their type compositions and are equally sized.” Recall that at the other extreme, ${N_C^{\circ }=1}$ only if communities are completely differentiated for their type compositions irrespective of the community sizes.

The latter suggests consideration of $N_C^{\circ }$ as indicating community differentiation provided the state of the absence of community differentiation includes equal community sizes to make the absence of differentiation “complete.” This inclusion makes sense when variable community sizes can be argued to have an ecological impact. In fact, it is hard to conceive a community ecological scenario in which community sizes and their variability are irrelevant. Maintaining this extended concept of differentiation, it follows that

$N_C^{\circ }$*varies from 1 to* $N_C,$*while structural characteristics vary from complete differentiation to complete absence of differentiation among communities*.

When compared with the observed number of communities, $N_C^{\circ }$ thus displays its twofold nature as a minimum number of communities and as an indicator of the amount of differentiation among communities.

Characterization of the structural aspects encoded in $N_{T<C}^{e}$ must be traced back to $N_C^{\circ }$ because of the “leading” function of the latter quantity. For example, $N_{T<C}^{e}=1$ is equivalent to $N_C^{\circ }=v_{TC}^e$, and this excludes any variation in types within and between communities. The metacommunity therefore consists of a single type (is monomorphic), which is in accordance with an effective number of types equal to 1. To assess the other extreme of $N_{T<C}^{e}$, i.e., $N_{T<C}^{e}=N_T$, let *N* again be the largest natural number with $v({{{\varvec{\mathsf{{ q}}}}}}^N_C)\le v_{TC}$ so that $v^e({{{\varvec{\mathsf{{ q}}}}}}_C^N)\le N\cdot N_T\le N_C^{\circ }\cdot N_T=v_{TC}^e$. In the case of $N_C^{\circ }=N$, the chain of inequalities implies that $v_{TC}^e=v^e({{{\varvec{\mathsf{{ q}}}}}}_C^N)\le N\cdot N_T=v_{TC}^e$ and $v^e({{{\varvec{\mathsf{{ q}}}}}}_C^N)=N\cdot N_T$, with the consequence that the marginal type distribution must be uniform. Since for given $v_{TC}$ and marginal type frequencies, any change in $N_C^{\circ }$ goes along with a change in $N_{T<C}^{e}$ but not in *N*, one infers that generally $N_{T<C}^{e}=N_T$ only if the marginal type distribution is uniform. Therefore,

*while* $N_{T<C}^{e}$*varies from 1 to* $N_T,$*the marginal type frequencies start with monomorphism and end with maximum polymorphism* (where all types are equally frequent).

This result is obvious for Rényi-diversities, since there always $N_{T<C}^{e}=v^e_T$.

The structural characteristics inherent in the factorization of joint diversity illustrate more vividly the conceptual advantages of the present approach, which focuses on the numbers of communities and types that essentially determine metacommunity diversity. By “essential,” two issues are addressed, factors and their interaction. The factors are specified by the minimally required number of communities (which depends on their differences in type composition) and the associated number of types (effectively represented in the minimally required number of communities). Their interaction is “orthogonal” in the sense of an independent operation in producing the joint diversity.

The factorization reminds of the common approaches to partitioning diversity in such a way that the total (metacommunity) type diversity $\gamma$ results as a product of the type diversity within communities ($\alpha$) with a quantity referred to as (type) “diversity” between communities ($\beta$), i.e., $\gamma =\alpha \cdot \beta$ (see, e.g., Jost 2007). This parallelism asks for pointing out potential relationships between the two approaches with special reference to the diversity within ($\alpha$) and between ($\beta$) communities as these do not explicitly appear in the factorization.

### $\alpha$- and $\beta$-diversity

Chiu et al. (2014, eq. (6)) introduced an alternative concept of $\alpha$-diversity for Rényi-diversities (Hill numbers) that relies on joint diversities and is termed “the effective number of species per assemblage” (types per community). It is treated within the frame of diversity partitioning. To show how their concept relates to the present factorization of joint diversity, note that for Rényi-diversities $v^e({{{\varvec{\mathsf{{ q}}}}}}^N_C )=v_T^e\cdot N$, and this is set equal to $v_{TC}^e$ to obtain $N_C^{\circ }=v_{TC}^e/v_T^e$. Since $N_C^{\circ }\le N_C$, one arrives at $v_{TC}^e/N_C\le v_T^e$, where $v_{TC}^e/N_C$ is argued by Chiu et al. (2014) to replace the classical versions of $\alpha$-diversity, i.e.,

$$\begin{aligned} \alpha =\alpha '=v_{TC}^e/N_C \end{aligned}$$

Since $v_{TC}^e\le N_T\cdot N_C$, one has $\alpha '\le N_T$, which is meaningful. However, if all communities are monomorphic, $v_{TC}^e=v_C^e$, and this allows for $\alpha '=v_C^e/N_C<1$. The interpretation of $\alpha '$ as “number of species per assemblage” should therefore not be misunderstood to indicate some kind of average of the diversities within communities (as applies to classical $\alpha$), since such averages are always greater or equal to 1 for effective numbers. Yet, as is demonstrated in Chao and Chiu (2016), $\alpha '$ has a broader application including variance decomposition approaches.

In particular, $v_T^e$ equals $\gamma$-diversity, so that $\alpha '\le \gamma$ and $\beta =\beta '=\gamma /\alpha '=N_C\cdot v_T^e/v_{TC}^e$. For Rényi-diversity $N_C^{\circ }=v_{TC}^e/v_T^e$, which yields

$$\begin{aligned} \beta '=N_C/N_C^{\circ }, \end{aligned}$$

and thus reveals the direct connection to the present factorization approach. It follows that $\beta '\le N_C$ with equality only for complete community differentiation, as it comes close to conventional views of partitioning total diversity ($\gamma$) into its component within ($\alpha$) and between ($\beta$) communities. The authors refer to their $\beta '$ as an “effective number of...completely distinct assemblages.”

Transferring the general properties of $N_C^{\circ }$ to $\beta '=N_C/N_C^{\circ }$, it becomes apparent that only in the complete absence of community differentiation (in the extended sense of equality of relative type frequencies among communities and equal community sizes) is $\beta '=1$. Variability in community sizes always implies $\beta '>1$, and $\beta '$ assumes its maximum of $N_C$ for complete community differentiation only. Chiu et al. (2014) arrived at a similar result for the special case of Rényi-diversities, where complete absence of community differentiation appears as “all assemblages are identical in species absolute abundances” (p.26).

Considering these relationships, it is tempting to accept the definition of $\alpha '=v_{TC}^e/N_C$ by Chiu et al. (2014) as a general approach (applying to all diversity measures), and see how this would fit into the common $\alpha$-$\beta$-$\gamma$ frame. Indeed, replacing $\gamma$ (the marginal diversity effective number of types) by $N_{T<C}^{e}$ (the associated effective number of types) one obtains $\beta '=\gamma /\alpha '=N_{T<C}^{e}/(v_{TC}^e/N_C)=N_{T<C}^{e}/(N_{T<C}^{e}\cdot N_C^{\circ }/N_C)=N_C/N_C^{\circ }$. Again $\gamma \ge \alpha '$ with equality, however, only in the complete absence of community differentiation.

Replacement of $\gamma$ by $N_{T<C}^{e}$ is justified in the first place by the observation that in the classical approach to partitioning diversity (relying on averaged diversities within communities and where $\gamma =v_T^e$), effects of type-community associations as taken account of in the joint diversity are not explicitly considered. On the other hand, the example of Rényi-diversity (where $N_{T<C}^{e}=v_T^e$) confirms the existence of families of diversity measures, for which effects of association can be captured in one component ($N_C^{\circ }$), while the other component ($N_{T<C}^{e}$) maintains its appearance as a marginal diversity. This characterizes the relationships between the present approach of factorizing joint diversity and the common $\alpha$-$\beta$-$\gamma$ approach to the partitioning of (type-)diversity.

The fact that the thus generalized $\beta '$ varies between 1 and $N_C$ and, because it equals $N_C$ only for completely differentiated communities, again reminds of the habit to interpret $\beta$ as an effective number of distinct communities. Yet, the above general reasoning brought forth against quantifying differentiation in terms of numbers of distinct communities is not remedied by the present $\beta '$. The numerical example in Table 2 demonstrates that each of the individual communities may be clearly differentiated from the others without any of them being completely distinct. In the common interpretation, the values of $\beta '$ in Table 2 would suggest that more than half of the three communities ($\beta ' >1.7$) are “completely distinct.” Distinctness of two among three communities, however, implies that all three are distinct, which contradicts ${\beta '<3}$.

There is actually no need to invoke numbers of distinct communities. The present $\beta '$ simply reverts the minimum characteristic of $N_C^{\circ }$ so that $\beta '$*specifies the maximally possible number of communities that realize the observed joint diversity given the observed trait distribution*. The maximum becomes 1 ($\beta '=1$) in the complete absence of differentiation (including equal community sizes) and reaches its largest value ($\beta '=N_C$) for complete differentiation. Even in the case of complete differentiation, it would be questionable to address $\beta '=N_C$ as an effective number of “distinct communities,” since this interpretation ignores unequal community sizes so that the same number of communities may apply to the case where one of 10 communities covers 99% of the metacommunity or where all 10 communities have equal share.

To allow for comparison across data sets, normalizations of $\beta '$ such as $(\beta '-1)/(N_C-1)$ or $(N_C-N_C^{\circ })/(N_C-1)$ may be required. Their interpretation is analogous, with the difference that the latter normalization is $N_C^{\circ }$ times the former. The two indices are of the general type where the realized value of a variable is relativized with respect to its minimum and maximum value. In the first case, the variable is $\beta '$ in the second $N_C^{\circ }$. Both indices can be interpreted as indices of dissimilarity among the communities (for further such indices of the Sørensen, Jaccard, or Morisity type, see, e.g., Chao et al. 2019, Table 1). The absence of type diversities or effective numbers of types in all of these indices is conspicuous. It makes apparent that the information on type distributions that is relevant in the assessment of structural aspects of community differentiation inherent in joint diversities can be summarized in a single quantity, namely $N_C^{\circ }$. However, remember that $N_C^{\circ }$ is conditional on the observed type frequencies.

### The type-centered perspective

Transition to the dual analogue shifts the focus to the type variable as leading factor with associated effective number of communities. The implied change from the community-centered to the type-centered perspective concentrates on the minimum number $N_T^{\circ }$ of types that is required to produce the observed metacommunity diversity measured by its joint diversity and given the observed community sizes. To appreciate the significance of this analogue, it is again helpful to take a look at the structural information that can be extracted from $N_T^{\circ }$ by considering the difference between $N_T$ and $N_T^{\circ }$.

Now, $N_T^{\circ }=1$ only if all types are completely differentiated for their community affiliations, which is tantamount to monomorphism of all communities. Herewith, different communities may be monomorphic for the same type or for different types. Conversely, $N_T^{\circ }$ reaches its maximum of $N_T$ only in the absence of differentiation of types for their community affiliations (type differentiation) together with equal frequencies of all types, i.e., for “complete” absence of type differentiation. Thus, as $N_T^{\circ }$ moves from 1 to $N_T$, structural characteristics start with complete type differentiation and thus monomorphism of communities and tend toward complete absence of type differentiation.

In an analogous manner, one concludes that the associated number $N_{C<T}^{e}$ of communities equals 1 if only one community exists, and it equals $N_C$ only if all communities have equal size.

These demonstrations suggest application of the type-centered perspective to the above-treated method of partitioning diversity proposed by Chiu et al. (2014). $\gamma$ then becomes $\gamma _d=N_{C<T}^{e}$, $\alpha '$ becomes $\alpha _d=v_{TC}^e/N_T$, and $\beta '$ becomes $\beta _d=N_T/N_T^{\circ }$. For Rényi-diversity, the corresponding quantities are $\gamma _d=v_C^e$, $\alpha _d=v_{TC}^e/N_T$, and $\beta _d=N_T\cdot v_C^e/v_{TC}^e$. Now, $\beta _d$ equals the maximally possible number of types that realize the observed joint diversity given the observed community sizes.

The unfamiliar type-centered perspective can be transferred into more familiar perceptions by considering that for given community sizes, the joint diversity decreases, and thus, $\beta _d$ increases, as the communities become less polymorphic. The evolutionary and ecological connotations then become apparent when realizing the consequences, for example, of competitive exclusion, drift under isolation, endemism or specialization. All of these are characterized by tendencies toward monomorphism. The opposite, i.e., the complete absence of monomorphism and thus complete polymorphism, implies equal type frequencies in addition to the absence of community differentiation. “Complete” absence of community differentiation is not relevant here, since community sizes are arbitrarily fixed observations.

In a more general context, systems of social or biochemical interaction that imply self- or cross-incompatibility within, between or across communities may directly influence type polymorphisms and their associations with communities. Herewith, self-incompatibility as coded by S-allele systems or realized in heterotypic mating or other heterotypic preferences, for example, tend to stabilize, balance and increase polymorphism, while systems of cross-incompatibility (such as heterozygote disadvantage, homotypic mating or other homotypic preferences, including inbreeding) destabilize polymorphisms and by this imply tendencies toward monomorphism.

Since in self-incompatibility systems, heterotypic combinations are preferred irrespective of community affiliations, potential differences in community affiliation between types tend to be equalized. This situation is characterized by small $\beta _d$-values ($N_T^{\circ }$ close to $N_T$) implying high polymorphism within communities. In the same manner, cross-incompatibility tends to enhance differences in community affiliation between types so as to prevent incompatible contacts within the same community. $\beta _d$-values are large in this case and imply low polymorphism within communities. From a generalized incompatibility point of view, $\beta _d$ can therefore be conceived of as reflecting effects on type diversity as are exerted by the continuum of compatibility reactions ranging between complete self- and cross-incompatibility.

### Comparison between the two perspectives

It remains to demonstrate how the two perspectives are connected. Since both perspectives rest on the same joint diversity $v_{TC}$, one obtains $N_{C<T}^{e}/N_{T<C}^{e}=N_C^{\circ }/N_T^{\circ }$, so that the ratio between numbers of communities and numbers of types is the same for associated effective numbers and for minimum numbers. Among other relationships between numbers of types and numbers of communities, such as $\gamma /\gamma _d=N_T^{\circ }/N_C^{\circ }$ and $\alpha '/\alpha _d=N_T/N_C$, it is interesting to see how the same numbers differ between perspectives. In this case, comparisons are to be made between minimum and associated numbers. Taking $N_C^{\circ }$ and $N_{C<T}^{e}$, a brief glance at the values in Table 2 informs us that the minimum numbers are all smaller than the associated numbers. The same holds for the number of types $N_T^{\circ }$ and $N_{T<C}^{e}$.

The latter observation becomes evident when considering the fact that $N_C^{\circ }/N_{C<T}^{e}=N_T^{\circ }/N_{T<C}^{e}=N_C^{\circ }\cdot N_T^{\circ }/v_{TC}^e$. Since $N_C^{\circ }$ and $N_T^{\circ }$ are minimum numbers based on the respective marginal distributions, one expects that $N_C^{\circ }\cdot N_T^{\circ }\le v_{TC}^e$ holds in general. For Rényi-diversities, where $N_C^{\circ }\cdot N_T^{\circ }/v_{TC}^e=v_{TC}^e/(v_T^e\cdot v_C^e)$, it is known that the inequality indeed applies for order ${a=1}$. Yet, the inequality does not extend to orders ${a\ne 1}$ (Gregorius 2010). Hence, in general the quotient is inappropriate for quantifying structural aspects of joint distributions. This confirms that structural characteristics of metacommunities should be assessed separately and independently between the community- and the type-centered perspective.

### Indeterminate (latent) community subdivisions

Often, the spatial distribution of communities does not show a sufficient degree of fragmentation to allow for determination of a unique subdivision into subcommunities. As a consequence, more than one subdivision may become relevant, each of which may show a different partitioning of the same underlying overall type diversity. Similarly, different criteria for subdivision, such as the spatial distribution of environmental variables, may be relevant in comparative causal analyses of observed genetic or species variation. In this case, the states of each variable give rise to a subdivision into “communities,” which in turn enables comparison of the effects of the various variables on the observed type variation.

The relevant studies are typically of the kind known from the analysis of latent variables (or factors), which is established especially in population genetics and implemented in methods such as structure (Pritchard et al. 2000), baps (Corander et al. 2003), or geneland (Guillot et al. 2005). When adapted to the present situation, these methods proceed from assignments of the observed individuals to hypothetical communities, where the assignments can be generated by one model, and a second model associates the community affiliations of the individuals with their types. The result of each such assignment is evaluated for fulfillment of special qualification criteria. The aim is to find assignments or functions thereof that optimize the qualification (for a demonstration of the general concept of the model-based analysis of latent causal factors see Gregorius 2018).

An assignment of the above kind specifies a joint distribution of types and community affiliations and therefore allows computation of the joint diversity together with its factorization into $N_C^{\circ }$ and $N_{T<C}^{e}$. Here, the associated effective number of types $N_{T<C}^{e}$ is of only secondary concern, since the marginal type distribution does not vary with the assignments. Particularly for Rényi-diversities, $N_{T<C}^{e}$ is unaffected by the assignments, since it equals the marginal type diversity. Thus, $N_C^{\circ }$, $N_C$ and $v_{TC}$ retain their significance as indicators of structural characteristics when applied to the assignments. In particular, $N_C^{\circ }$ increases strictly with $v_{TC}$ because of the invariance of the marginal type distribution.

In most cases, the models underlying an analysis of latent factors are based on probability laws that specify likelihoods or posterior probabilities of the assignments. These probabilities serve as the primary qualification criterion that is to be maximized (especially maximizing likelihood). This allows consideration of the most likely structural characteristics indicated by $N_C^{\circ }$ and $N_C$ and their relations (in terms of $\beta '$, for example). In many applications, however, such as the methods cited above, the probability laws are used in MCMC-algorithms to estimate the expected values of the indicators, instead of the posterior probabilities of assignments as subdivisions of all individuals into communities.

## Footnotes

1 In view of the popularity of Rényi-diversities (see Table 1), it might be useful to recall the significance of diversity measures whose effective numbers need not be Rényi-diversities. In many cases, Rényi-diversities result from diversity measures that reflect models of random encounter (see, e.g., Patil and Taillie 1982). Encounters can, however, be non-random with preferences especially for types other than the own. Such encounter probabilities could be of the form $(1-p)^a$ with *p* as type frequency, where for $0<a<1$, the encounter would take place more frequently than at random. Tendencies to encounter other types more frequently than at random are commonly conceived of as cross-preference. Hence, $Q:=\sum _ip_i\cdot (1-p_i)^a$ is the average cross-preference. It is straightforward to show that $p\cdot (1-p)^a$ is a concave function of *p* for $0<a\le 1$, which was demonstrated by Gregorius (2014) to imply validity of the evenness criterion for *Q* and by this confirms *Q* to be a measure of diversity. The effective number of this measure becomes $1/(1-Q^{1/a})$, which, except of ${a=1}$, is not a Rényi-diversity.
